# Evaluation of Triclosan Effects on Cultured Swine Luteal Cells

**DOI:** 10.3390/ani11030606

**Published:** 2021-02-25

**Authors:** Giuseppina Basini, Simona Bussolati, Simone Bertini, Fausto Quintavalla, Francesca Grasselli

**Affiliations:** Dipartimento di Scienze Medico-Veterinarie, Università degli Studi di Parma, Via del Taglio 10, 43126 Parma, Italy; simona.bussolati@unipr.it (S.B.); simone.bertini@unipr.it (S.B.); fausto.quintavalla@unipr.it (F.Q.); francesca.grasselli@unipr.it (F.G.)

**Keywords:** corpus luteum, progesterone, nitric oxide, superoxide anion, redox status

## Abstract

**Simple Summary:**

A great concern has been raised against many chemicals, both natural and man-made, that can mimic or interfere with the hormones. Among these, using swine ovarian cells, we were aimed to explore the potential effect of triclosan, an antimicrobial agent widely used in cosmetics and home products. Our results demonstrate that triclosan disrupts cellular function, in particular interfering with hormone production and proliferation, thus suggesting a critical evaluation of its effects.

**Abstract:**

Triclosan is a chlorinated phenolic, used in many personal and home care products for its powerful antimicrobial effect. Several studies have shown triclosan toxicity and the American Food and Drug Administration (FDA) in 2016 has limited its use. It has been recently included in endocrine-disrupting chemicals (EDCs), a list of chemicals known for their ability to interfere with hormonal signaling with particular critical effects on reproduction both in animals and humans. In order to deepen the knowledge in this specific field, the present study was undertaken to explore the effect of different concentrations of triclosan (1, 10, and 50 µM) on cultured luteal cells, isolated from swine ovaries, evaluating effects on growth Bromodeoxyuridine (BrDU) incorporation and Adenosine TriPhosphate (ATP) production, steroidogenesis (progesterone secretion) and redox status (superoxide and nitric oxide production, enzymatic and non-enzymatic scavenging activity). A biphasic effect was exerted by triclosan on P4 production. In fact, the highest concentration inhibited, while the others stimulated P4 production (*p* < 0.05). Triclosan significantly inhibited cell proliferation, metabolic activity, and enzymatic scavenger activity (*p* < 0.05). On the contrary, nitric oxide production was significantly increased by triclosan (*p* < 0.01), while superoxide anion generation and non-enzymatic scavenging activity were unaffected.

## 1. Introduction

Triclosan (5-chloro-2-(2,4-dichlorophenoxyl)-phenol) is a powerful synthetic, chlorinated phenolic antimicrobial and antifungal agent found in almost all toothpastes, soaps, creams, mouthwashes, dishwashing liquids. Moreover, triclosan can be added to other materials, such as textiles or plastics to make them resistant to bacterial growth [1]. Thus, the exposure results from the use of consumer products containing triclosan, and small amounts of the substance can be absorbed through the skin or the mouth. Unfortunately, due to its chemical structure, triclosan can accumulate in adipose tissue and its concentration can rise due to biomagnification [2]. It has been detected in human blood (0.01–38 ng/mL), urine (2.4–3790 μg/L), and breast milk (100–2100 μg/kg) [3,4,5]. Numerous studies have shown its toxicity [6,7,8] and the American Food and Drug Administration (FDA) in 2016 has finally limited its use [9]. Triclosan has been associated with an increased risk of cancer development [10] and it has also been demonstrated to negatively affect intestinal microbiota [11] and to determine antibiotic resistance occurrence [12]. Recently, triclosan has been included in endocrine-disrupting chemicals (EDCs), a list of chemicals known for their ability to interfere with hormone action resulting in adverse health consequences in animals and humans. In particular, negative effects on male [13] and female [14] reproduction have been suggested. Corpus luteum is a transient structure that develops after ovulation and its correct function is vital for pregnancy, and, in general, for ovarian cycle [15]. Therefore, we aimed to explore the effects of 1, 10, and 50 µM triclosan [16] on cultured swine luteal cell viability, proliferation, progesterone production, and redox status parameters [17]. Pigs were chosen as experimental animals since, due to their similarities with humans, results can be used for translational medicine [18].

## 2. Materials and Methods

All reagents used in this study were obtained from Sigma (St. Louis, MO, USA) unless otherwise specified.

### 2.1. Isolation of Luteal Cells

Swine ovaries were collected at a local slaughterhouse from Large White cross-bred gilts, parity = 0. The days of the estrous cycle were unknown, so it was only possible to perform evaluations based on the ovarian morphology using previously validated methods [19,20,21,22,23]. Ovaries from 20 animals evaluated to be in luteal phase were placed into sterile phosphate-buffered saline (PBS, 4 °C) supplemented with 500 IU/mL penicillin, 500 µg/mL streptomycin, and amphotericin B (3.5 µg/mL) [24,25,26] and transported to the laboratory within 1 h. To improve cleaning, the ovaries were then immersed for 1 min in ethanol 70% and washed with sterile PBS. Luteal tissue, obtained from pools of freshly excised corpora lutea of ten animals in mid-luteal phase of the estrous cycle, was enzymatically dissociated according to the technique of Gospodarowicz and Gospodarowicz [27]. Corpora lutea were minced opportunely and luteal cells were enzymatically dissociated in PBS containing BSA (1 mg/mL), collagenase type-I and II (1 mg/mL; Gibco, Waltham, MA, USA), and DNAse (1 mg/mL) incubating 1 h at 37 °C in a shaker water bath [26]. Afterwards, cells were filtered through a 40 µm filter, treated with ammonium chloride 0.17 M at 37 °C for 1 min to remove red blood cells, and centrifuged at 300× *g* for 10 min. Cells viability was determined after vital staining with trypan blue dye (0.4% *w*/*v*).

### 2.2. Triclosan Effects on Cultured Luteal Cells

Luteal cells were plated into 96-well plates (Sarstedt, Nümbrecht, Germany) at different seeding densities in 200 µL of culture medium (CM) composed by M199 supplemented with sodium bicarbonate (2.2 mg/mL), penicillin (100 UI/mL), streptomycin (100 mg/mL), amphotericin B (2.5 mg/mL) and 10% fetal bovine serum (FBS) [26]. Plates were then incubated at 37 °C, 5% CO_2_, and 95% humidified air for 48 h, in the presence and absence of triclosan at the concentration 1, 10 and 50 µM triclosan [16].

#### 2.2.1. Luteal Cell Proliferation

Luteal cell proliferation was evaluated by BrdU incorporation assay test (Roche Diagnostics, Mannheim, Germany). Cells (10^4^/well) were seeded in 96-well plates and treated with triclosan at the concentrations indicated above. After 24 h of incubation (37 °C, 5% CO_2_, and 95% humidified air), BrdU (20 µL) was added to each well and incubated overnight. At the end, plates were centrifuged at 400× *g* for 10 min, CM was removed, FixDenat Solution (200 µL) was added to each well. The reaction was stopped, and the product was quantified by measuring absorbance at a wavelength of 450 nm with Victor Reader spectrophotometer (Perkin Elmer, Groningen, The Netherlands) [25].

#### 2.2.2. Luteal Cell Metabolic Activity

Viable cells (2 × 10^5^/well) were seeded in 96-well plates in 200 µL CM and treated with triclosan for 48 h as above indicated. Cell metabolic activity was assessed using a bioluminescent ATP assay (ATP-lites; Packard Bioscience, Groningen, The Netherlands) ATP, being present in all metabolically active cells, is a viability marker whose concentration declines very rapidly when the cells undergo either necrosis or apoptosis. The ATP lite-M assay system is based on the detection of light produced by the luciferase catalyzed reaction of ATP with D-luciferin. The emitted light is proportional to the ATP concentration. Briefly, 50 µL of mammalian cell lysis solution was added to 100 µL of cell suspension. Then 50 µL of substrate solution was added to the wells and the luminescence was measured by Victor reader.

#### 2.2.3. Luteal Cell Progesterone (P4) Production

Cells (10^4^/well) were seeded in 96-well plates and incubated with triclosan as above indicated for 48 h (37 °C, 5% CO_2_, and 95% humidified air). Plates were then centrifuged at 400× *g* for 10 min and supernatants were collected, frozen, and stored at −20 °C until P4 determination. P4 concentration, inversely proportional to the developed color intensity, was measured with Progesterone ELISA (DiaMetra, Boldon, UK) and using a Victor reader at a wavelength of 450 nm through a calibration curve [17].

#### 2.2.4. Luteal Cell Redox Status

##### Non-Enzymatic Scavenging Activity

The non-enzymatic scavenging activity was measured by the ferric reducing ability of plasma (FRAP) assay, which measures the reduction of ferric-tripiridyltriazine (Fe^3+^ TPTZ) into ferrous form (Fe^2+^ TPTZ), as previously reported [26]. TPTZ reagent was prepared before use, mixing 25 mL of acetate buffer, 2.5 mL of 2,4,6-Tris(2-pyridyl)-s-triazine (TPTZ) 10 mM in HCl 40 mM and FeCl_3_–6H_2_O solution. Cells (2 × 10^5^/well) were seeded in 96-well plates and treated with triclosan as indicated above for 48 h (37 °C, 5% CO_2_, and 95% humidified air). At the end, plates were centrifuged at 400× *g* for 10 min, supernatants were discarded, and cells were lysed, in ice bath for 30 min, with cold Triton 0.5% + PMSF in PBS (200 µL/well). 40 µL of cell lysates were added to Fe^3+^ TPTZ reagent and incubated at 37 °C for 30 min. The reduction developed a blue color that was read by Victor Reader at 595 nm. The reducing ability was determined using a standard curve of absorbance against FeSO_4_-7H_2_O standard solution.

##### Enzimatic Scavenging Activity: Superoxide Dismutase (SOD)

SOD activity was determined by a SOD Assay Kit (Dojindo Molecular Technologies, Kumamoto, Japan). Cells (2 × 10^5^/200 μL CM/well) were seeded in 96-well plates (Sarstedt, Nümbrecht, Germany) and treated for 48 h with triclosan as above detailed. After centrifugation for 10 min at 400× *g*, the surnatants were discarded and cells were lysed adding cold Triton 1% in TRIS HCl (100 µL/10^5^ cells) and incubating on ice for 30 min. Cell lysates were tested without dilution and a standard curve of SOD ranging from 0.156 to 20 U/mL was prepared. The absorbance was determined with Victor Reader reading at 450 nm against 620 nm.

##### Luteal Cell Nitric Oxide (NO) Production

Griess test was used to evaluate NO production by measuring nitrite levels in supernatants of cultured cells [26,28,29]. Cells (10^5^ cells/well) were seeded in 96-well plates and incubated with triclosan as above detailed for 48 h at 37 °C, 5% CO_2_, and 95% humidified air. Plates were then centrifuged for 10 min at 400× *g* and supernatants were collected. After 15 min incubation with Griess reagent, the absorbance was measured with Victor Reader using 540 nm against 620 nm filter.

##### Luteal Cell Superoxide (O_2_^−^) Production

Cell-proliferation reagent WST-1 test (Roche Diagnostics, Indianapolis, In, USA) was used to quantify O_2_^−^ production in cultured cells [25,30]. Cells (10^4^/well) were seeded in 96-well plates and treated with triclosan at the concentrations indicated above for 48 h (37 °C, 5% CO_2_, and 95% humidified air). WST-1 (20 µL) was added to each well during the last 4 h of incubation; at the end, absorbance was determined using Victor reader at a wavelength of 450 nm against 620 nm.

### 2.3. Statistical Analysis

The experiments were repeated six times with six replicates for each treatment. Data are presented as mean ± SEM (standard error of mean). Statistical difference was calculated by one-way ANOVA using Statgraphics software (STC Inc., Rockville, MD, USA). In the presence of a significant difference (*p* < 0.05), the means were subjected to Scheffé F test for multiple comparisons.

## 3. Results

### 3.1. Luteal Cell Proliferation

**Figure 1:**
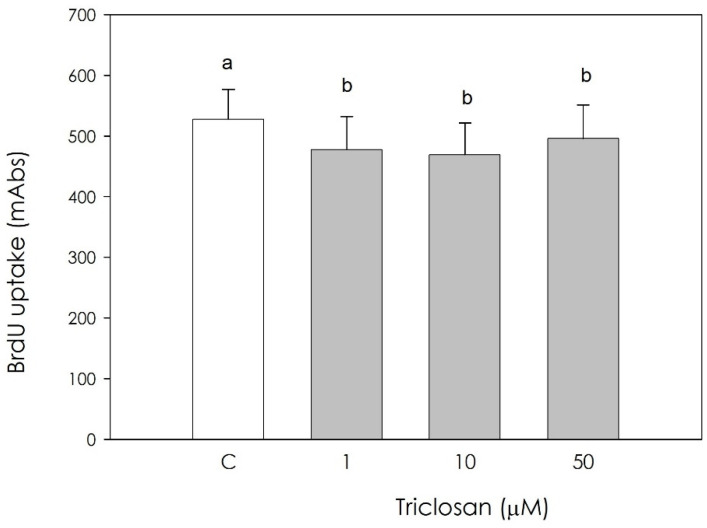
Effect of the treatment with triclosan (1, 10, or 50 µM) for 48h on swine luteal cell proliferation using 5-bromo-2′-deoxyuridine (BrdU) incorporation assay test. Data, expressed as milliAbs units, represent the mean ± SEM of 36 replicates. Different letters on the bars indicate a significant difference (*p* < 0.05) among treatments as calculated by one-way ANOVA and Scheffè’ F test.

Triclosan significantly inhibited (*p* < 0.05) cell proliferation (Figure 1), with no significant difference in treatment efficacy between all the examined concentrations.

### 3.2. Luteal Cell Metabolic Activity

**Figure 2:**
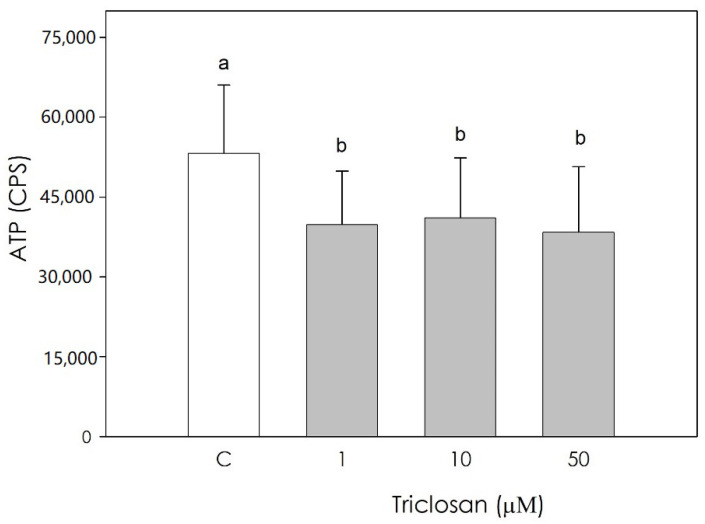
Effect of the treatment with triclosan (1, 10, or 50 µM) for 48 h on swine luteal cell metabolic activity using ATP assay test. Data, expressed as CPS, represent the mean ± SEM of 36 replicates. Different letters on the bars indicate a significant difference (*p* < 0.05) among treatments as calculated by one-way ANOVA and Scheffè’ F test.

Luteal cell metabolic activity, was significantly inhibited (*p* < 0.05) by all the examined triclosan concentrations without difference among them (*p* < 0.05) (Figure 2).

### 3.3. Luteal Cell Progesterone (P4) Production

**Figure 3:**
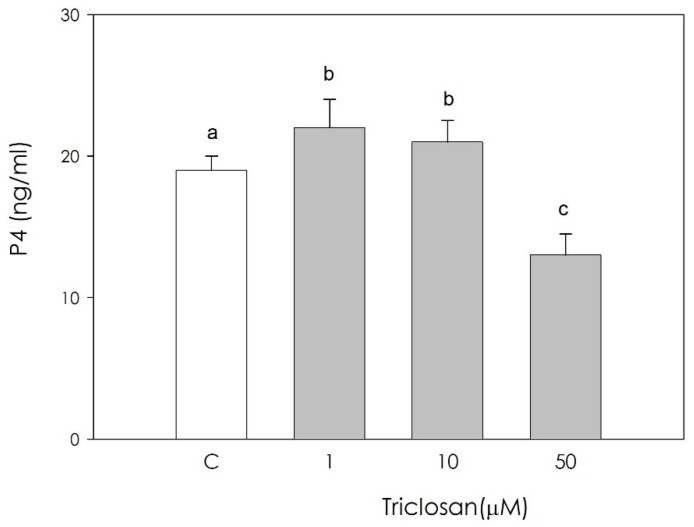
Effect of the treatment with triclosan (1, 10, or 50 µM) for 48 h on swine luteal cell progesterone (P4) production using ELISA assay. Data, expressed as ng/mL, represent the mean ± SEM of 36 replicates. Different letters on the bars indicate a significant difference (*p* < 0.05) among treatments as calculated by one-way ANOVA and Scheffè’ F test.

P4 output (Figure 3) was significantly inhibited (*p* < 0.05) by triclosan at the highest concentration tested while the other concentrations displayed a stimulatory effect (*p* < 0.05).

### 3.4. Luteal Cell Redox Status

Non-enzymatic scavenging activity was unaffected in luteal cells treated with triclosan. 

**Figure 4:**
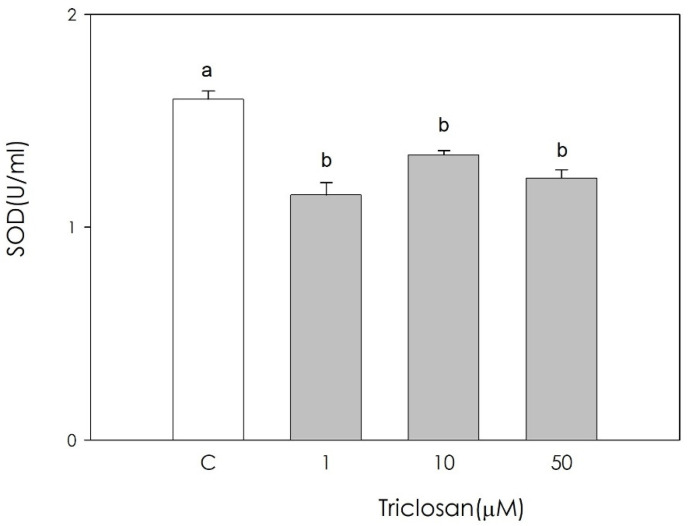
Effect of the treatment with triclosan (1, 10, or 50 µM) for 48 h on swine luteal cell enzymatic scavenging activity using the SOD assay. Data, expressed as U/mL, represent the mean ± SEM of 36 replicates. Different letters on the bars indicate a significant difference (*p* < 0.05) among treatments as calculated by one-way ANOVA and Scheffè’ F test.

On the contrary, enzymatic SOD activity was significantly inhibited by all the examined triclosan concentrations without difference among them (*p* < 0.05) (Figure 4).

**Figure 5:**
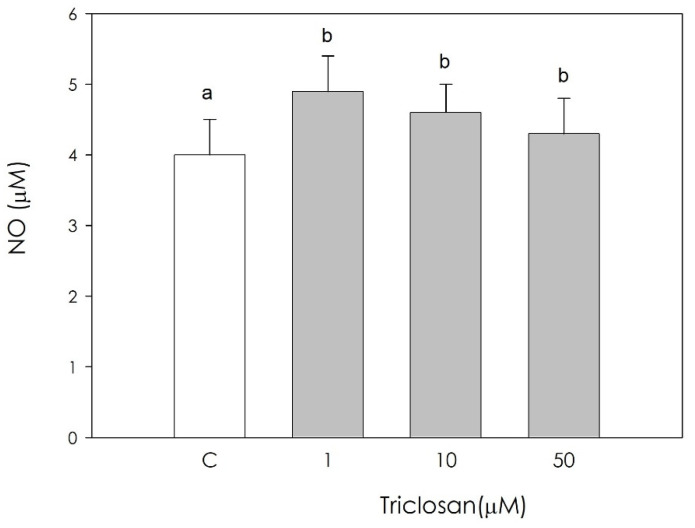
Effect of the treatment with triclosan (1, 10, or 50 µM) for 48 h on swine luteal cell nitric oxide (NO) production using Griess Assay. Data, expressed as µM, represent the mean ± SEM of 36 replicates. Different letters on the bars indicate a significant difference (*p* < 0.01) among treatments as calculated by one-way ANOVA and Scheffè’ F test.

NO production was significantly increased by triclosan. All the examined concentrations were equally effective (*p* < 0.01) (Figure 5).

O_2_^−^ generation resulted unaffected by triclosan, at all tested concentrations.

## 4. Discussion

Endocrine disruptors are natural or synthetic chemicals that can impair the normal hormone function by turning on/off or modifying the hormonal signaling, with resulting adverse effects in an organism [28,30,31,32,33,34,35,36]. Among EDCs, a great concern has been recently raised as regards to reproductive effects of triclosan, a chlorinated antimicrobial agent widely used in soaps, healthcare antiseptic scrubs, and some personal hygiene products (e.g., toothpaste, mouthwash, acne cream, deodorant, and lotions) [13]. However, despite growing evidence on its disrupting action on fertility, to the best of our knowledge, the effects have been never investigated on corpus luteum, a transient endocrine organ, which function is essential for a correct ovarian cyclicity and a successful pregnancy [37]. First of all, we studied triclosan potential interferences on luteal cell growth and metabolic activity [17,26], since other endocrine disruptors have been previously demonstrated to affect these functions in ovarian cells [35,36,38,39]. As for triclosan, no previous studies have been published about these topics in luteal cells. Jurewicz et al. [40] demonstrated a negative effect on ovarian follicle reserve, even if the mechanism of action has not been elucidated while Chen et al. [16] showed its inhibitory effects on rat granulosa cell viabilty. 

A crucial ovarian function is undoubtedly represented by steroidogenesis. The ovarian steroid hormones are essential for reproduction but are also involved in cardiovascular, central nervous, and skeletal muscle system functions. Progesterone is one of main hormonal ovarian product which pivotal roles in reproduction are well known. It is actually crucial in the establishment and maintenance of early pregnancy by determining the endometrial receptivity and transforming the endometrial stromal cells into enlarged, secretory cells. Thus, the failure in its action results in implantation failure and miscarriage. Corpus luteum represents the main site of progesterone production, a process which is tightly regulated and maintained by multiple factors, both systemic and local [41]. Growing evidence indicates that endocrine-disrupting chemicals interfere with ovarian steroidogenesis [34,35,36,39]. As for corpus luteum, Romani et al. [42,43] showed that both phenols and phthalates inhibited P4 production by human luteal cells. Moreover, phthalates (DEHP) inhibit P4 production also in rabbit corpus luteum, with a mechanism that seems to involve PPARG expression down-regulation, an increase of PTGS2 activity and prostaglandin F2alpha secretion, 3beta-HSD down-regulation [44]. To our knowledge, the effect of triclosan has never been tested on progesterone production by isolated luteal cells. Chen et al. [16] found that it stimulated P4 synthesis by rat granulosa cells. On the contrary, our present results on luteal cells show a biphasic effect of the molecule, with stimulatory effect at the lower concentrations and inhibition induced by the highest concentration tested. In general, this is not a novel finding when the effects of endocrine disruptors are tested [45]. Therefore, further studies are necessary to widen the range of tested concentrations and to unravel the mechanisms of triclosan disruptive action on P4 synthesis. 

It is well known that redox status is crucial for ovarian function. This balance can be altered by increased levels of reactive oxygen species (ROS) and/or reactive nitrogen species (RNS, i.e., nitric oxide, NO), as well as by a decrease in antioxidant defense mechanisms [46]. As regards to corpus luteum, ROS, RNS, and antioxidants have been recognized as key factors involved in its function, with special emphasis on steroidogenesis and new vessel growth regulation and finally in the definition of its demise [47]. To our knowledge, the effect of triclosan on redox status of luteal cells has never been explored. In general, endocrine disruptors have been found to affect ROS, RNS, and antioxidant balance in reproductive cells [48,49,50], but their effects in corpus luteum have not been sufficiently investigated. Our data show that O_2_^−^ generation and non-enzymatic scavenger activity were unaffected by triclosan. On the contrary, both SOD activity and NO production were modified, thus indicating a potential interference in the redox status balance. Moreover, the disruption of NO generation could have a detrimental effect in regard to the crucial role of this molecule in luteal angiogenesis [51]. It should be noted that the blood supply of the mature corpus luteum is the highest per-unit tissue of any body organ and new vessels develop under strict regulation [37]. Moreover, the increase in NO generation could be involved in P4 disruption, as recently reviewed in the rabbit model [52].

## 5. Conclusions

Taken together, present study demonstrates that triclosan interferes with the main function of cultured swine luteal cells thus suggesting that it can disrupt the physiological function of corpus luteum, a transient endocrine organ essential for a correct ovarian cyclicity and for a successful pregnancy.

## Data Availability

Data will be available upon request.
